# The contribution of cytogenetics and flow cytometry for understanding the karyotype evolution in three *Dorstenia* (Linnaeus, 1753) species (Moraceae)

**DOI:** 10.3897/CompCytogen.v10i1.6719

**Published:** 2016-01-22

**Authors:** Paulo Marcos Amaral-Silva, Wellington Ronildo Clarindo, Tatiana Tavares Carrijo, Carlos Roberto Carvalho, Milene Miranda Praça-Fontes

**Affiliations:** 1 Laboratório de Citogenética, Departamento de Biologia, Centro de Ciências Agrárias, Universidade Federal do Espírito Santo. CEP: 29.500-000 Alegre – ES, Brazil; 2 Laboratório de Botânica, Departamento de Biologia, Centro de Ciências Agrárias, Universidade Federal do Espírito Santo. CEP: 29.500-000 Alegre – ES, Brazil; 3 Laboratório de Citogenética e Citometria, Departamento de Biologia Geral, Centro de Ciências Biológicas e da Saúde, Universidade Federal de Viçosa. CEP: 36.570-000 Viçosa – MG, Brazil

**Keywords:** Cytogenetics, euploidy, karyotype, flow cytometry, chromosome structure

## Abstract

Chromosome morphometry and nuclear DNA content are useful data for cytotaxonomy and for understanding the evolutionary history of different taxa. However, the chromosome number is the only karyotype aspect reported for the species of *Dorstenia* so far. In this study, the nuclear genome size of *Dorstenia arifolia* (Lamarck, 1786), *Dorstenia bonijesu* (Carauta & C. Valente, 1983) and *Dorstenia elata* (Hooker, 1840) was evaluated and their karyotype morphometry accomplished, with the aim of verifying the potential of those parameters to understand evolutionary issues. Mean nuclear 2C value ranged from 2C = 3.49 picograms (pg) for *Dorstenia elata* to 2C = 5.47 pg for *Dorstenia arifolia*, a variation of ± 1.98 pg. Even though showing a marked difference in 2C value, the three species exhibited the same 2n = 32. Corroborating the flow cytometry data, differences in chromosome morphology were found among the karyotypes of the species investigated. Based on this and the only phylogeny proposed for *Dorstenia* thus far, structural rearrangements are related to the karyotype variations among the three species. Besides, the karyological analysis suggests a polyploid origin of the *Dorstenia* species studied here.

## Introduction

The pantropical genus *Dorstenia* Linnaeus, 1753 comprises about 105 species distributed in Asia, Africa and Neotropical regions (Carauta 1978, Berg 2001). Thirty-nine species occur in Brazil (Romaniuc-Neto et al. 2015), representing the sections *Dorstenia* Linnaeus, 1753, *Emigdioa* Carauta, 1976 and *Lecanium* Fischer & Meyer, 1846. Carauta (1978) and Berg (2001) have contributed much of the knowledge about the morphology of Neotropical species, while the phylogenetic reconstructions using molecular data (Misiewicz and Zerega 2012) consolidated the hypothesis about the genus’ monophyly. Despite the potential information of cytogenetic data for taxonomic (Stace 2000) and phylogenetic studies (Melo and Guerra 2003), this marker has been little explored in previous works on *Dorstenia*.

According to the current knowledge, the basic chromosome number in African species of *Dorstenia* is x = 12 and x = 13, while in American species x = 14, 15 and 16 (Berg 2001) are found. Fagerlind (1944) reported *Dorstenia mannii* Hooker f., 1871 as a tetraploid (2n = 48). In addition, intraspecific variation in chromosome number have been reported for subspecies of *Dorstenia psilurus* Welwitsch, 1869 (2n = 26 and 2n = 40) (Misiewicz and Zerega 2012) and *Dorstenia elata* Hooker, 1840 2n = 26 or 32 (Krause 1931, Hoen 1983, unpublished data). These data indicate that the evolution of the karyotype in *Dorstenia* involved euploidy and aneuploidy events. However, it is still necessary to invest efforts in confirming and understanding the chromosome changes reported for some species in previous studies. Considering that chromosome alterations are a significant mechanism of diversification and speciation in Angiosperms (Stace 2000, Peer et al. 2009, Weiss-Schneeweiss et al. 2013), the investigation of this aspect in *Dorstenia* can bring light to the knowledge of speciation processes in the genus.

Cytogenetic approaches, which regard the chromosome number, morphometric measurements and karyotype analysis, contribute to the understanding of evolutionary processes in plants (Shan et al. 2003). The knowledge of these aspects in related species helps to elucidate issues related to diversification of a taxonomic group (Clarindo et al. 2012). Morphometric analysis of the chromosomes is a way to determine the karyotype changes that occurred throughout evolution, the processes that led to the diversification, and the direction taken by evolution (Gao et al. 2012).

Numeric and structural chromosome rearrangements have been reported as triggers of karyotype changes in several plant taxa. Therefore, nuclear genome size variation occurs between phylogenetically related species due to these alterations (Bonifácio et al. 2012, Raskina et al. 2008). For this reason, nuclear DNA content measurement has been increasingly employed in systematic approaches using flow cytometry (FCM). In addition to its practicality and reproducibility, FCM is useful to reveal differences between taxa (Stace 2000), especially in groups of species that exhibit conserved chromosome number (Mabuchi et al. 2005).

The cytogenetic and FCM approaches in *Dorstenia* could provide relevant information to sections and species taxonomy, as well as contribute to understanding the evolutionary history of the genus. The main goal of this study was therefore to measure the nuclear 2C value, determine the chromosome number and characterize the karyotype of Neotropical species of *Dorstenia*: *Dorstenia arifolia* Lamarck, 1786, *Dorstenia bonijesu* Carauta & C. Valente, 1983 and *Dorstenia elata* Hooker, 1840.

## Material and methods

**Figure 1.**
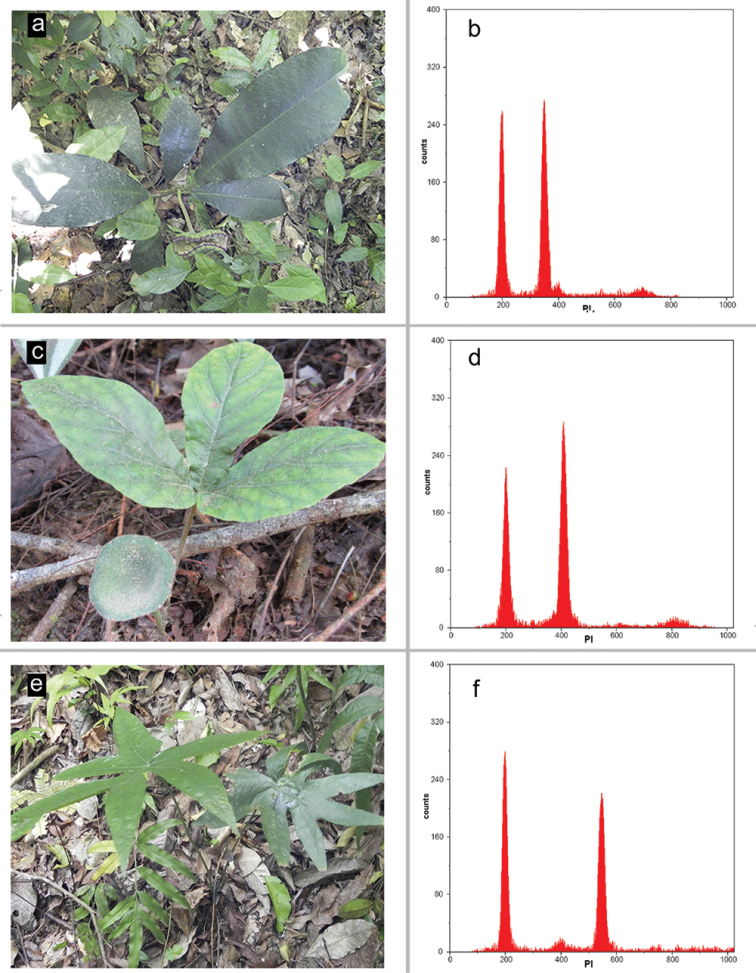
Representative adult plants of *Dorstenia elata* (**a**), *Dorstenia bonijesu* (**c**) and *Dorstenia arifolia* (**e**) of the Atlantic Rainforest remnant located in the Castelo city (ES, Brazil). FCM histograms showing G_0_/G_1_ peaks generated by nuclei suspensions of *Solanum lycopersicum* (internal standard, channel 200, 2C = 2.00 pg), and of *Dorstenia elata* (**b** channel 349, 2C = 3.49 pg), *Dorstenia bonijesu* (**d** channel 405, 2C = 4.05 pg) and *Dorstenia arifolia* (**f** channel 547, 2C = 5.47 pg).

Plant samples – Specimens of *Dorstenia elata*, *Dorstenia bonijesu* and *Dorstenia arifolia* (Fig. 1 a, c, e, respectively) were collected in an Atlantic Rainforest remnant located in the city of Castelo – ES, Brazil. Voucher specimens were included in the herbarium VIES: T.T. Carrijo 1516 (*Dorstenia arifolia*); T.T. Carrijo 1682 (*Dorstenia bonijesu*) and T.T. Carrijo 1618 (*Dorstenia elata*).

FCM – Nuclear suspensions were obtained by chopping (Galbraith et al. 1983) of leaf fragments (1 cm^2^) excised from each *Dorstenia* species (sample) and *Solanum lycopersicum* Linnaeus, 1753 (internal standard, 2C = 2.00 picograms – Praça-Fontes et al. 2011). Samples and standard leaf fragments were simultaneously chopped with razor blade in a Petri dish containing 0.5 ml OTTO-I nuclear extraction buffer (Otto 1990) supplemented with 2 mM dithiothreitol and 50 µg ml^-1^ RNAse (Praça-Fontes et al. 2011). Afterwards, 0.5 ml of the OTTO-I buffer was added, the suspensions were filtered through 30-µm nylon mesh, placed into microtube and centrifuged at 100 × *g* for 5 min. The precipitate was resuspended in 100 µl OTTO-I buffer and incubated for 10 min (Praça-Fontes et al. 2011). The nuclei suspensions were stained with 1.5 ml of OTTO-I:OTTO-II (1:2 – Otto 1990, Loureiro et al. 2006) solution supplemented with 2 mM dithiothreitol, 50 µg ml^-1^ propidium iodide and 50 µg ml^-1^ RNAse (Praça-Fontes et al. 2011). The nucleus suspensions were kept in the dark for 30 min, then filtered through 20-µm nylon mesh. The samples were analyzed in a flow cytometer Partec PAS II/III (Partec GmbH, Germany). Histograms were analyzed with the Partec Flow Max software tools to measure nuclear DNA content. The genome size of the *Dorstenia* species was calculated according to the formula:


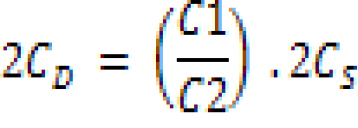


wherein:

2C_D_: value of 2C DNA content (pg) of each *Dorstenia* species,

C1: average G_0_/G_1_ peak channel of the *Dorstenia* species,

C2: average G_0_/G_1_ peak channel of *Solanum lycopersicum*,

2C_S_: value of 2C DNA content of *Solanum lycopersicum* (2.00 pg).

Cytogenetics – Stems with length of approximately 15 cm exhibiting one leaf pair were excised and disinfected with 1% NaOCl_2_ solution for 15 min. These propagules were maintained in hydroponic system for rooting. The system was oxygenated by a compressor coupled to a plastic hose, which was immersed in H_2_O. The roots were treated with 3 µM or 4 µM amiprophos-methyl (APM) for 16 h or 18 h at 4 °C. The roots were washed in dH_2_O for 20 min, fixed in methanol:acetic acid solution (3:1) and stored at -20 °C (Carvalho et al. 2007). After 24 h, the roots were washed in dH_2_O and macerated in pectinase solution 1:40, 1:45, 1:50, 1:55 or 1:60 (enzyme:dH_2_O) for 1 h 45 min or 2 h at 34 °C. The material was washed in dH_2_O, fixed again and kept at -20 °C until use. Using the macerated root meristems, slides were prepared by cell dissociation and air-drying techniques, then placed onto a hot plate at 50 °C (Carvalho et al. 2007). The slides were stained with 5% Giemsa (Merck®) for 8 min, washed twice in dH_2_O, and air-dried. All slides were analyzed under a Nikon Eclipse Ci-S microscope (Nikon). The capture of metaphase images was performed using 100× objective and a CCD camera (Nikon Evolution^TM^) coupled to a Nikon 80i microscope (Nikon).

Morphometric analysis – The chromosomes of three *Dorstenia* species were characterized as to the total length, length of the long and short arms, and classes. The chromosome class was determined as proposed by Levan et al. (1964) and reviewed by Guerra (1986): r = length of the long arm / length of the short arm. The asymmetry of the karyotype was evaluated using the method proposed by Zarco (1986), using the formulae:


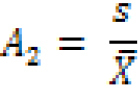


where A_2_ = interchromosomal asymmetry index, s = standard deviation, and X = average length of the chromosomes.

## Results

### Flow cytometry measurements

The nuclear suspensions resulted in histograms with G_0_/G_1_ peaks showing coefficient of variation below 3.45%. Thus, the isolation and staining procedures provided suspensions containing isolated, intact and stoichiometrically stained nuclei. Using the histograms, the mean 2C nuclear DNA content of *Dorstenia* species was measured for the first time. The values were 2C = 3.49 pg ± 0.0035 (1C = 1.71 bp × 10^9^) for *Dorstenia elata*; 2C = 4.05 pg ± 0.014 (1C = 1.98 bp × 10^9^) for *Dorstenia bonijesu*; and 2C = 5.47 pg ± 0.002 (1C = 2.67 bp × 10^9^) for *Dorstenia arifolia* (Fig. 1 b, d, f). The mean value for *Dorstenia arifolia* was 36.20% higher than for *Dorstenia elata*, and 26.00% greater than for *Dorstenia bonijesu*. Besides, *Dorstenia bonijesu* showed a DNA content 13.83% higher than that of *Dorstenia elata*. Based on these values, an interspecific variation of the nuclear genome size was identified between the species.

### Cytogenetics

**Figure 2.**
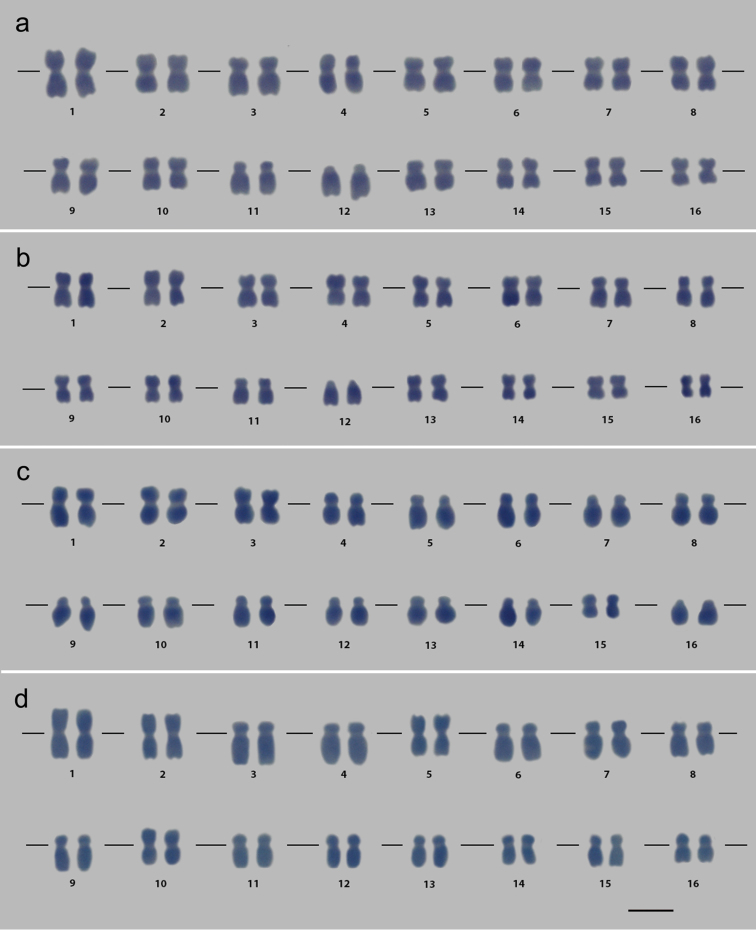
Karyograms assembled from mitotic chromosomes of *Dorstenia elata* (**a**, **b**), *Dorstenia bonijesu* (**c**) and *Dorstenia arifolia* (**d**). Note the distinct chromatin compact level (**a**, **b**) showed by the mitotic chromosomes of *Dorstenia elata*. **a**, **b** *Dorstenia elata* showed twelve metacentric chromosome pairs (1, 2, 4, 5, 7, 8, 9, 10, 13, 14, 15 and 16), two submetacentric (3 and 6) and two acrocentric pair (11 and 12) **c** *Dorstenia bonijesu* exhibited four metacentric chromosome pairs (1, 2, 3 and 15), ten submetacentric (4, 5, 6, 8, 9, 10, 11, 12, 13 and 14) and two acrocentric ones (7 and 16) **d** *Dorstenia arifolia* presented four metacentric chromosome pairs (1, 2, 5 and 10) and twelve submetacentric ones (3, 4, 6, 7, 8, 9, 11, 12, 13, 14, 15 and 16). Bar: 5 μm.

The rooting of vegetative propagules occurred after 40 days in hydroponic system. The disinfection of the propagules contributed for relatively rapid rooting due to absence of contamination. Owing to the lack of cytogenetic studies for the genus *Dorstenia*, we tested different treatments with microtubule inhibitor as well as distinct procedures of enzymatic maceration. Root meristems treated with 4 µM APM for 16 h at 4 °C and macerated in 1:60 pectinase solution for 1 h 45 min at 34 °C resulted in metaphases adequate for karyotype characterization of the *Dorstenia* species (Fig. 2).

The differences chromatin compaction levels verified among the metaphases (Fig. 2 a, b) of the species allowed morphometric analysis (Table 1) and karyogram assembly (Fig. 2). All *Dorstenia* species showed a conserved number of 32 chromosomes.

**Table 1.** Morphometry of the metaphasic chromosomes of *Dorstenia elata* (2C = 3.49 pg, 2n = 32), *Dorstenia bonijesu* (2C = 4.05 pg, 2n = 32) and *Dorstenia arifolia* (2C = 5.47 pg, 2n = 32).

Chrom.	*Dorstenia elata*						*Dorstenia bonijesu*						*Dorstenia arifolia*					
	Total (µm)	Arms		r	Class	Size (%)	Total (µm)	Arms		r	Class	Size (%)	Total (µm)	Arms		r	Class	Size (%)
		Short	Long					Short	Long					Short	Long			
1	4.72	2.09	2.63	1.26	M	8.01	4.50	1.95	2.55	1.31	M	7.97	5.39	2.57	2.82	1.10	M	7.93
2	4.27	2.00	2.27	1.14	M	7.25	4.27	1.90	2.37	1.25	M	7.56	5.21	2.45	2.76	1.13	M	7.67
3	4.21	1.66	2.55	1.54	SM	7.15	4.04	1.81	2.23	1.23	M	7.15	4.87	1.48	3.39	2.29	SM	7.17
4	3.87	1.78	2.09	1.17	M	6.57	4.00	1.45	2.55	1.76	SM	7.08	4.81	1.39	3.42	2.46	SM	7.08
5	3.84	1.75	2.09	1.19	M	6.52	3.86	1.27	2.59	2.04	SM	6.83	4.69	2.27	2.42	1.07	M	6.90
6	3.81	1.48	2.33	1.57	SM	6.47	3.81	1.45	2.36	1.63	SM	6.75	4.36	1.42	2.94	2.07	SM	6.42
7	3.81	1.69	2.12	1.25	M	6.47	3.72	0.90	2.82	3.13	A	6.59	4.33	1.39	2.94	2.12	SM	6.37
8	3.72	1.57	2.15	1.37	M	6.32	3.59	1.22	2.37	1.94	SM	6.36	4.18	1.45	2.73	1.88	SM	6.15
9	3.66	1.54	2.12	1.38	M	6.21	3.59	1.22	2.37	1.94	SM	6.36	4.18	1.27	2.91	2.29	SM	6.15
10	3.59	1.75	1.84	1.05	M	6.10	3.54	1.00	2.54	2.54	SM	6.27	3.93	1.81	2.12	1.17	M	5.78
11	3.54	0.71	2.83	3.99	A	6.01	3.40	0.95	2.45	2.58	SM	6.02	3.93	1.36	2.57	1.89	SM	5.78
12	3.48	0.62	2.86	4.61	A	5.91	3.04	0.86	2.18	2.53	SM	5.38	3.84	1.39	2.45	1.76	SM	5.65
13	3.30	1.33	1.97	1.48	M	5.60	2.95	0.95	2.00	2.11	SM	5.22	3.78	1.21	2.57	2.12	SM	5.56
14	3.24	1.42	1.82	1.28	M	5.50	2.95	0.81	2.14	2.64	SM	5.22	3.63	1.18	2.45	2.08	SM	5.34
15	3.03	1.30	1.73	1.33	M	5.14	2.63	1.09	1.54	1.41	M	4.66	3.51	1.24	2.27	1.83	SM	5.16
16	2.81	1.36	1.45	1.07	M	4.77	2.59	0.59	2.00	3.39	A	4.59	3.33	1.21	2.12	1.75	SM	4.90
Sum	56.09	24.36	34.54	-	-	100.00	53.89	19.42	37.06	-	-	100.00	64.64	25.09	42.88	-	-	100.00

Chrom – chromosome; r – arm ratio (long/short); Size – % size in relation to sum of the mean values of total length; M – metacentric; SM – submetacentric; A – acrocentric; Sum – sum of the mean values.

### Morphometric analysis of the chromosomes

The mean values for the sum of total length as well as short- and long-arm length differed among the species (Table 1). *Dorstenia arifolia* showed the highest total, short- and long-arm length in relation to other species, corroborating the FCM data. *Dorstenia bonijesu*, which exhibited an intermediate mean 2C value, presented the lowest total chromosome and short-arm length. In comparison to *Dorstenia elata*, *Dorstenia bonijesu* displayed a greater mean value for the long arm (Table 1). The A2 index also varied between the species: *Dorstenia bonijesu* exhibited the most asymmetrical karyotype, with A_2_ = 0.16, followed by *Dorstenia arifolia* (A_2_ = 0.14) and *Dorstenia elata* (A_2_ = 0.13).

Based on the morphometric data, the chromosome class was determined and the differences between the karyotypes for the three species were endorsed. *Dorstenia elata* showed twelve metacentric chromosome pairs (1, 2, 4, 5, 7, 8, 9, 10, 13, 14, 15 and 16), two submetacentric (3 and 6) and two acrocentric pair (11 and 12) (Fig. 2 a, b). *Dorstenia bonijesu* exhibited four metacentric chromosome pairs (1, 2, 3 and 15), ten submetacentric (4, 5, 6, 8, 9, 10, 11, 12, 13 and 14) and two acrocentric ones (7 and 16) (Fig. 2c). *Dorstenia arifolia* presented four metacentric chromosome pairs (1, 2, 5 and 10) and twelve submetacentric ones (3, 4, 6, 7, 8, 9, 11, 12, 13, 14, 15 and 16) (Fig. 2d).

## Discussion

Despite exhibiting the same number of chromosomes (2n = 32), the species of *Dorstenia* studied here show distinct mean nuclear 2C values. The interspecific DNA content variation indicates that the karyotypes differ between the species (Gitaí et al. 2014). Considering that the *Dorstenia* species have the same chromosome number, the karyotype divergences are probably associated to chromosome structure.

The morphometric analysis revealed karyomorphological differences in the sum of the mean values for total chromosome length, and the short arm and long arm (Table 1). Besides, some chromosomes presented distinct classes among the species, such as in chromosomes 7 and 16, which are submetacentric in *Dorstenia arifolia*, acrocentric in *Dorstenia bonijesu* and metacentric in *Dorstenia elata* (Fig. 2, Table 1). In view of this, structural chromosomal rearrangements have occurred during the evolutionary history of the group. This is supported by the interchromosomal asymmetry index (A_2_, Zarco 1986), which also varied between the three *Dorstenia* species, despite the predominance of metacentric and submetacentric chromosomes. Therefore, the FCM and cytogenetic data indicate that structural chromosome changes have occurred throughout the evolution of these *Dorstenia* species.

According to the more recent phylogeny for the genus, *Dorstenia arifolia* occupies a basal position in comparison to *Dorstenia elata* (Misiewicz and Zerega 2012). Given that *Dorstenia arifolia* has mean nuclear 2C value and total genome length (Fig. 1 and 2, Table 1) greater than *Dorstenia elata*, the structural chromosome alterations seem to promote loss of DNA sequences. Thus, deletions can be involved in the karyotype changes in *Dorstenia*.

Based on the morphometric analysis, groups of morphologically identical chromosome pairs were found for each *Dorstenia* species: 3–4, 6–7, 11–12 and 13–14 in *Dorstenia arifolia*; 5–6, 11–12 and 13–14 in *Dorstenia bonijesu*; and 7–8 and 14–15 in *Dorstenia elata* (Fig. 2, Table 1). Regarding this karyotype aspect, polyploidization events have occurred during the evolution of these species. As the three species produces reduced reproductive cells (x = 16), the disploidy can also explain the evolutionary scenario of the *Dorstenia* karyotype. The polyploid origin of plant species has been shown by classical cytogenetics, such as for the genera *Psidium* Linnaeus, 1753 (Souza et al. 2015), *Claytonia* Linnaeus, 1753 (McIntyre 2012) and *Cardamine* Linnaeus, 1753 (Marhold et al. 2010). From the assembly of accurate karyograms using chromosomes with different levels of chromatin compaction, the type of polyploidy was also evidenced, with autopolyploidy being found in *Glycine max* (Linnaeus, 1753) Merrill, 1917 (Clarindo et al. 2007) and *Zephyranthes* Herbert, 1821 (Felix et al. 2011), and allopolyploidy in *Paullinia cupana* Kunth, 1821 (Freitas et al. 2007) and *Triticum aestivum* Linnaeus, 1753 (Kamel 2006). Polyploidy has played a key role in plant evolution, with estimates maintaining euploidy in the ascendancy of all angiosperms (Abbott et al. 2013, Soltis et al. 2014). In addition, 15% of speciation events in this taxon are directly involved with polyploidization (Abbott et al. 2013).

## Conclusion

The nuclear 2C value and karyogram indicate changes covering chromosome number and structure that occurred during the karyotype evolution of *Dorstenia arifolia*, *Dorstenia bonijesu* and *Dorstenia elata*. The combination of FCM and classical cytogenetics revealed differences among the *Dorstenia* species that can be exploited in phylogenetic approaches, as the results support the current knowledge on the phylogeny of *Dorstenia*.
